# A Note on Congenital Dilatation of the Ureters with Hydronephrosis

**Published:** 1905-09

**Authors:** J. M. Fortescue-Brickdale

**Affiliations:** Physician to Out-Patients and Pathologist at the Bristol Royal Hospital for Sick Children and Women


### A NOTE ON CONGENITAL DILATATION OF THE URETERS WITH HYDRONEPHROSIS.

BY

J. M. Fortescue-Brickdale, M.A., M.D. Oxon.,

*Physician to Out-Patients and Pathologist at the Bristol Royal Hospital for Sick Children and Women.*

---

During the past twelve months I have seen three specimens of this curious condition, two of which were exhibited at the Society for the Study of Disease in Children and one at the Bristol Medico-Chirurgical Society. None of the children affected showed any symptoms of hydronephrosis during life, and that condition can only have been a contributing factor to their death. The ages of the children were five months, one year, and two years and two months respectively. All the children were wasted. Two died of acute bronchitis and one of suppurating spina bifida. Other congenital deformities are said by Dr. Emmett Holt to be rare in these cases, but Mr. Henry Morris seems to hold a contrary opinion.

The anatomical conditions found *post mortem* in all three cases were practically identical, differing only in degree. The most marked case was that in the child dying youngest, but this was the one complicated by spina bifida.

In all three there was marked hypertrophy of the bladder; the ureters were dilated and tortuous, and the kidneys hydronephrotic. In all of them a catheter was easily passed *post mortem*, and no evidence of a urethral valve or other cause of obstruction to the urethra could be discovered on dissection. There was no phimosis. The dilatation of the ureters ended abruptly at the bladder walls, the intra-muscular portion being very small in calibre.

The entire urinary system may easily be removed *en masse* by the following method: The kidneys and ureters are dissected off the posterior abdominal wall, and the dissection carried as far down round the base of the bladder as possible. A

catheter is then passed into the bladder to mark the urethra, the symphysis pubis is divided, and a portion of the cartilage removed. The mucous membrane of the prepuce is then snipped round close to the corona glandis, and the penis is pulled back into the abdomen. It is then easily removed without damaging the skin of the organ, which may be stuffed with a little cotton-wool.

I have remarked that the intra-muscular part of the ureters is always very narrow, and in the last specimen examined I found that though urine could easily be squeezed out of the bladder through the urethra, it was impossible to force water from the ureter into the bladder. I therefore came to the conclusion that the primary fault lay in the bladder walls, the hypertrophied condition of which may be compared to that of the pylorus in congenital hypertrophic stenosis. I regard the condition, in fact, as a primary hyperplasia of the bladder walls, due possibly to disordered nervous influence. To this is superadded, I believe, an element of muscular spasm, for the condition of the orifices of the ureters, like that of the hyperplastic pylorus in some instances, cannot be one of continuous atresia. Urine was passed in all my cases during life; but the spasmodic contractions of the thickened muscle compressing the orifices of the ureters for longer or shorter periods are sufficient to account for the backward pressure on the ureters and kidneys. It is not unlikely that irregularities in the micturition of a young infant should be overlooked, and had careful observations been made symptoms pointing to hydronephrosis might perhaps have been observed in all these cases.

The microscopical appearances of the tissues do not throw much additional light on the ætiology of the condition, chronic nephritis more or less advanced, with dilatation of the urinary tubules, being commonly found.

It cannot, of course, be said that primary hyperplasia of the bladder is the cause of dilated ureters in all infantile cases. Various anatomical obstructions have been found in the urethra and elsewhere in some cases. In some cases there is no marked thickening of the bladder walls, and in others a definite valve is found in the urethra. An interesting case of the last-named kind was reported by Mr. Morton at the meeting of the Society for the Study of Disease in Children, held in Bristol in 1904, where the valve, which admitted a No. 3 catheter, offered considerable obstruction to urine flowing in an opposite direction.

Dr. Holt has reported ten cases, in only three of which a definite obstruction was found in the urethra; and Drs. Bryant and Hale White have described a most remarkable case in which there was also obliterative endarteritis with calcification of the adventitia, the child being only six months old. In this case a very extreme degree of phimosis was present, and was considered sufficient to account for the condition of the ureters and kidney. In some of Dr. Holt's cases the condition was unilateral, and in the bilateral cases some difference in degree was generally observed between the two sides. This, if hyperplasia of the bladder is present, or some obstruction is found lower down in the urinary tract, may be accounted for by differences in the activity of the two kidneys. In some of Dr. Holt's cases microscopical changes were found in both kidneys when only one ureter was dilated.

There does not seem to be much evidence of any close relationship between this condition and congenital cystic kidney. In one of Dr. Holt's unilateral cases a cystic condition of the other kidney was observed, most probably as a result of the nephritis, and not developmental in origin. Backward pressure of urine is much more likely to lead to hydronephrosis, as is remarked by Mr. Morris.

The condition may be regarded as rare, but would probably be found more often if it were more generally looked for, as has certainly been the case with other pathological states to which attention has recently been drawn.

### REFERENCES.

HOLT. *Diseases of Children*, Ed. altera, p, 651.
MORTON. *Rep. Soc. Study Dis. Child.*, 1903-04, iv. 329.
BRYANT and HALE WHITE. *Guy's Hosp. Rep.*, 1901, lv. 17.
MORRIS. Allbutt's *System of Medicine*, vol. iv. 1897, p. 452.

